# The Rising Burden of Childhood Obesity: Prevention Should Start in Primary School

**DOI:** 10.3390/nu17040650

**Published:** 2025-02-11

**Authors:** Debora Porri, Malgorzata Wasniewska, Giovanni Luppino, Letteria Anna Morabito, Elisa La Rosa, Giorgia Pepe, Domenico Corica, Mariella Valenzise, Maria Francesca Messina, Giuseppina Zirilli, Alessandra Li Pomi, Aurora Lanzafame, Angela Alibrandi, Tommaso Aversa

**Affiliations:** 1 Department of Human Pathology of Adulthood and Childhood, University of Messina, Via Consolare Valeria, 98124 Messina, Italy; debora.porri@gmail.com (D.P.); malgorzata.wasniewska@unime.it (M.W.); giorgia.pepe@unime.it (G.P.); domenico.corica@unime.it (D.C.); mariella.valenzise@unime.it (M.V.); francesca.messina@unime.it (M.F.M.); giuseppina.zirilli@polime.it (G.Z.); alessandra.lipomi92@gmail.com (A.L.P.); auroralanzafamemd@gmail.com (A.L.); tommaso.aversa@unime.it (T.A.); 2 Pediatric Unit, “G. Martino” University Hospital, 98122 Messina, Italy; letteria.morabito@gmail.com (L.A.M.); elisalarosa@icloud.com (E.L.R.); 3 Department of Economics, University of Messina, 98100 Messina, Italy; angela.alibrandi@unime.it

**Keywords:** childhood obesity, childhood obesity prevention, pediatric nutrition, lifestyle intervention, Mediterranean diet, Kid-Med questionnaire

## Abstract

**Background:** The increasing rates of childhood obesity (CO) are an ongoing problem. We focused on the adherence to the Mediterranean diet (MD), physical activity, and sleep habits of preschool children in order to investigate the relationship between lifestyle habits and health outcomes through parental perception. **Methods:** In the context of “EpPOI: Education to prevent CO”, we investigated physical activity (PA) and sleep hygiene using an online survey for caregivers. Parents also completed the Mediterranean Diet Quality Index for children and adolescents (Kid-Med) questionnaire. **Results:** A total of 5.3% of the interviewees achieved a score indicating an adequate adherence to the MD. Additionally, 50.5% of children ate sweets every day, and 80% skipped breakfast. We also found that the parents’ perceptions of their children’s PA were a predictor of MD adherence, and PA was also positively correlated with fruit consumption [*p* < 0.034], vegetable consumption [*p* < 0.015], and fish consumption [*p* < 0.005]. **Conclusions**: Parents seem to be oriented towards a healthy lifestyle, but the children’s level of adherence to the Mediterranean diet is poor. CO prevention requires a synergic effort that includes an adherence to healthy eating patterns such as the MD and a greater awareness of parents on the importance of adopting a healthy lifestyle at an early age.

## 1. Introduction

Childhood obesity (CO) represents a critical public health challenge, with its prevalence steadily rising globally [1]. The worldwide trend shows that about 41 million children under the age of 5 years are overweight, and 340 million children and adolescents aged 5 to 19 years are affected by obesity [2]. In our region of study, Italy, the prevalence of overweight and obesity in children aged 7–13 years reached 28.2% and 12.2%, respectively, in 2016 [3]. The onset of obesity starts at an early age; a recent review highlighted the need to understand the causes of obesity in preschool children in view of the long-term detrimental consequences of obesity [4]. To address this problem, a multidimensional approach focused on modifiable lifestyle factors is needed early on in life. Different dimensions of an individual’s lifestyle contribute, even in a synergic way, to the development of childhood obesity. Poor nutrition and physical inactivity have been found to play a key role, but recent evidence also underlines the importance of sleep [5,6,7]. For instance, the COVID-19 pandemic significantly disrupted daily routines, contributing to an alarming increase in CO rates worldwide. A recent Italian study [8] highlighted how the COVID-19 pandemic negatively affected lifestyle habits; to reinforce this consideration, the data collected in a study by Valenzise and colleagues [9] confirmed an increase in sedentary behavior and the establishment of unhealthy eating habits. Lockdowns and school closures resulted in reduced physical activity, irregular sleep patterns, and poor sleep hygiene, factors closely associated with weight gain in children. A study published by Ochoa-Moreno et al. revealed a 45% increase in obesity rates among primary school-aged children in the UK during the 2020–2021 lockdown period, highlighting the profound impact of these behavioral changes [10]. Prolonged inactivity due to limited opportunities for outdoor play and structured exercise compounded this problem. The shift to remote learning further limited opportunities for movement, replacing physical education classes and active recess periods with prolonged sedentary hours in front of screens. A longitudinal study conducted in Italy [11] showed that children’s average daily physical activity dropped by 50%, while screen time nearly doubled compared to pre-pandemic levels. In the same study, an increase in the consumption of unhealthy foods such as snacks and sugary drinks was observed, along with a decrease in the overall quality of the participants’ diet. Similar results were found by Di Renzo et al. [12], who found that there was an increased consumption of junk food with consequent weight gain.

In addition, in the above-mentioned study [11], the authors also found a significant alteration in sleep–wake rhythms, where participants showed a change in sleep rhythms, with a tendency to go to sleep later and wake up later.

The pandemic’s influence on daily routines includes reduced physical activity and increased screen time. The effects of physical activity on obesity could be very important; several pieces of evidence suggest that increased physical activity reduces the risk of obesity in preschool children [13]. At the same time, increased screen time in place of physical activity reduces energy expenditure and disrupts circadian rhythms, contributing to a shorter sleep duration and poorer sleep quality [14]. Recent evidence has underscored the link between sleep disturbances and metabolic dysregulation in children, which heightens the risk of obesity; thus, adequate sleep is a critical yet often overlooked component of obesity prevention. Sleep deprivation affects metabolic processes, appetite regulation, and energy balance, which can predispose children to weight gain [15]. In a cross-sectional study of 48,922 3-year-old children, the authors found an association between sleep duration and a higher risk of overweight/obesity at an early age [16]. Recent studies emphasize the importance of reducing sedentary behaviors, such as television watching and the recreational use of electronic devices, as essential preventive measures. For instance, high screen time has been linked to a 38% increase in the risk of obesity across various age groups, underscoring its impact on weight management strategies [17]. Although new technologies could be useful in the fight against childhood obesity [18], parental awareness regarding their use in children is fundamental.

A pillar of effective CO prevention is the adherence to a Mediterranean diet (MD), which promotes a balanced intake of fruits, vegetables, whole grains, and healthy fats [19]. This dietary pattern, characterized by a high consumption of vegetables and fruits, whole grains, legumes, fish, and olive oil, with a moderate intake of dairy and meats, has been linked to a reduced risk of overweight and obesity in children [20]. Recent studies highlight that a greater adherence to the MD is associated with a lower body mass index (BMI) in children and a decreased prevalence of obesity, attributed to its balanced nutritional profile rich in bioactive compounds with anti-inflammatory and antioxidant properties [21,22]. Promoting nutritional education and fostering adherence to this dietary model could play a pivotal role in mitigating the prevalence of CO globally. Evidence shows that children are more likely to adopt healthier eating behaviors when parents consistently model such habits, serving as a primary model for food preferences and consumption patterns [23].

Public health interventions can effectively target CO by integrating the following strategies: reducing sedentary behaviors, ensuring adequate sleep, and promoting a Mediterranean dietary pattern by strengthening parental skills and encouraging positive role modeling. This multifaceted approach aligns with evidence-based practices and provides a sustainable framework for improving child health outcomes. The EpPOI project builds on these core concepts [24], underlining the role of the schools, which must guarantee a favorable environment where healthy habits are built. The overall project aims to identify the gaps to be filled in order to implement the effective and early prevention of childhood obesity in preschool-aged children, with the ultimate goal of providing specific models to promote healthier lifestyles at an early age.

This last study specifically aimed to investigate the relationship between lifestyle habits and health outcomes by focusing on the following three key aspects: adherence to an MD, quality and duration of sleep, and levels of physical activity, assessed through the parents' perceptions of their children's lifestyles.

## 2. Materials and Methods

The present study is part of the “EpPoi—Educare per Prevenire l’Obesità Infantile” project, aimed at raising awareness about the risk of childhood obesity [24]. We focused on the age group of kindergarten children (4–5 years) at Ex csa 4^ Istituto Comprensivo Leopardi, in Messina, where previous interventions with school staff were carried out on dedicated days during the school timetable for data recording.

An anonymous online survey for parents was administered using Google Forms, and the data were downloaded as a Microsoft Excel sheet. In addition to the demographic information, we also investigated aspects related to physical activity and sleep hygiene.

Regarding physical activity, we asked the parents, “How physically active do you think your child is?”, in the form of a Likert scale structured as follows: 1 = very little; 2 = a little; 3 = quite a lot; 4 = a lot; 5 = extremely.

We also asked whether the child performs structured activities. To assess sleep hygiene, we asked whether their child sleeps more or less than 7 hours a night, if he or she takes an afternoon nap, and when he or she uses electronic devices, in the form of a Likert scale where 1 indicates “a little” and 5 indicates “a lot”.

To evaluate the adherence to an MD, we administered the Mediterranean Diet Quality Index for children and adolescents (Kid-Med) questionnaire to the parents [25].

This tool is based on a 16-question test that can be either be self-administered or conducted through an interview. Questions reflecting a negative connotation regarding adherence to the MD are assigned a score of -1, while those indicating a positive aspect are scored at +1. The total score from the test is used to classify subjects into the following three levels of adherence to the Mediterranean diet:

1. Score ≥ 8: Optimal adherence to the MD;
2. Score 4–7: Moderate adherence, with improvements needed to align dietary habits with Mediterranean patterns;
3. Score ≤ 3: Poor adherence, indicating very low diet quality.

The Kid-Med test is an effective tool to assess the quality of food habits [26]. Participants were fully instructed about the study aim and were also informed that by agreeing to fill in the questionnaire, they confirmed their participation, automatically providing informed consent. There were no direct benefits to the respondents from participating in this study.

## 3. Statistical Analysis

### 3.1. Power and Sample Size Calculation

Assuming a two-tailed test for correlation between the variables, a medium effect size equal to 0.3 [27], and a significance level of 5%, the minimum number of subjects to be enrolled for the study to guarantee a statistical power of 80% was found to be 82 subjects. An invitation to participate in the survey was then sent to 100 families and, among these, the total number of respondents was 95; this sample size guaranteed 80% power.

### 3.2. Data Analysis

Categorical variables were expressed as absolute frequencies and percentages, while numerical variables were expressed as mean ± standard deviation (SD), minimum and maximum.

The Kolmogorov–Smirnov test was applied in order to check the possible normal distribution of numerical variables; consequently, since we verified the Gaussian distribution for the numerical data, a parametric approach was used for data analysis.

A cumulative proportional odds model was estimated to assess the possible dependence of the MD adherence variable on other possible predictors such as the children’s sleep hours and parents’ perceptions of their children’s physical activity (how physically active do you think your child is?), adjusted for age and educational status.

A *p*-value lower than 0.050 was considered statistically significant.

All statistical analyses were performed by using SPSS for Windows package, version 22.

## 4. Results

A total of 95 subjects completed the survey and were all mothers. The percentage frequencies relating to the demographic variables (age and level of education) of the respondents are summarized in Table 1.

Approximately 62.8% of mothers declared that their child slept more than seven hours a night, while 37.2% reported that their child slept less than seven hours. Additionally, more than half of the respondents (53.8%) confirmed that their child took naps. Concerning electronic device utilization, the percentage frequencies are reported in Table 2.

Table 3 shows the percentage frequencies of the responses to the question, “How physically active do you think your child is?”. A majority of the respondents gave the maximum score of 5; therefore, they considered the child to be physically active.

Unfortunately, only 5.3% of the interviewees reached a score ≥8 on the Kid-Med questionnaire, which refers to an optimal adherence to the MD. A high percentage of the respondents (47.4%) reached a point of 3, which indicates a low adherence to the MD. Results from the Kid-Med questionnaire are reported in Table 4, expressed as frequency and percentage. Respectively, 47.4% and 44.2% of the interviewees obtained a score indicating poor and medium adherence to the MD.

We also analyzed the responses to each individual question on the Kid-Med questionnaire. Notably, 55.7% of parents declared that their child regularly consumed vegetables, and 71% consumed at least one fruit a day. A percentage of 93.7% used olive oil, while a percentage higher than half of the sample (62.1%) consumed legumes at least once a week. Unfortunately, 50.5% of children consumed sweets and candy several times every day, and 80% skipped breakfast. Answers to the individual Kid-Med items are reported in Table 5. Out of a sample of 95 subjects, 3 did not fill out the entire questionnaire, while 1 that was identified as “missing” avoided answering some questions.

To conduct an in-depth analysis, we evaluated the correlation between the lifestyle variables of interest. No correlations were found between the sleep habits, expressed as hours of sleep per night of less or more than 7, and the use of devices, as well as between the sleep habits and the Kid-Med scores. Moreover, the children’s physical activity was positively correlated with the Kid-Med score [*p* < 0.024].

Further analysis of the individual Kid-Med items revealed a positive correlation between the parents’ perceptions of their children’s physical activity and the following items:

“Takes fruit every day?” [*p* < 0.034];

“has fresh or cooked vegetables regularly once a day?” [*p* < 0.015];

“consumes fish regularly (at least 2–3 times/week)?” [*p* < 0.005].

We also found a negative correlation between the parents’ perceptions of their children’s physical activity and the item, “takes candy or sweets several times every day?” [*p* < 0.26].

We performed an ordinal regression to identify the predictors of good adherence to the Mediterranean diet, and we found the parental perception of their child’s physical activity as a predictor. As expressed in Table 6, this result was confirmed in the model adjusted for age and education level.

## 5. Discussion

Childhood obesity is a significant public health issue worldwide, and its prevalence continues to rise despite numerous preventive efforts [28]. Nutrition, particularly adherence to healthy dietary patterns like the Mediterranean diet (MD), plays a crucial role in mitigating this trend. The MD is characterized by a high consumption of fruits, vegetables, whole grains, legumes, nuts, and olive oil, with a moderate intake of fish, and dairy, and a low consumption of red meat and sweets. A recent meta-analysis by Grosso et al. [29] emphasized that the adherence to an MD is inversely associated with obesity in children and adolescents, primarily due to its nutrient density and anti-inflammatory properties. A recent study [30] highlighted some factors that were positively associated with a better adherence to the MD and, among these, there was also regular physical activity. This aspect was also confirmed in our study, considering the positive correlation found between the caregivers’ perception of their child’s physical activity and some elements related to MD, in particular fruit and vegetable consumption and the regular consumption of fish. However, despite these positive dietary trends, the overall adherence score to the MD was very low, with only 5.3% of respondents reaching a score ≥ 8 on the Kid-Med score, which indicates an adequate adherence. Our results are in line with the other works that have evaluated MD adherence. For instance, Sanlier et al. [31] also found that preschooler MD adherence was below the optimal level, by using the same version of the Kid-Med questionnaire. The Kid-Med questionnaire has been extensively used to assess MD adherence, particularly in Europe [32], and recent evidence suggests a decline in the adherence to an MD among children from Mediterranean countries, according to this scoring method [26,32,33]. This decline may be partially attributed to the increasing globalization of dietary habits, which introduces higher intakes of processed foods and sugary beverages [34].

Intervention programs targeting dietary adherence have shown varying success rates, often depending on their scope and target population; a recent meta-analysis suggested that interventions conducted outside of school targeting both children and caregivers and focusing on participants with overweight or obesity are more effective in promoting optimal adherence to the Mediterranean diet [35], probably due to the presence of caregivers.

It is worth noting that our data on the children’s habits were entirely provided by their parents. While this may be considered a limitation, it also offers significant value, as the family environment plays a crucial role in shaping the children’s eating behaviors, which are largely acquired within the household. Moreover, the dynamic relationships among parental perceptions, education, and children’s dietary habits introduce additional complexity to addressing childhood obesity prevention [36]. It has been known for a long time that the parental perception of diet quality is influenced by many different social, economic, biological, and psychological factors, and a parent’s perception of their child’s diet may not accurately reflect this reality [37]. Another limitation was that the respondents were all mothers, and this may have contributed to gender bias. A recent study conducted as part of the European Childhood Obesity Project (EU CHOP) [38] on both mothers and fathers of 432 eight-year-old children showed no overall differences between the mothers and fathers in rating their child’s weight status, despite a propensity for underestimation. Altered parental/caregiver perception remains an issue that needs to be addressed. In another recent study with a large sample, the authors examined the level of parental perception with a 5-point Likert scale, the same method as ours. They noted that the concordance between parental perception and actual weight status improved from 2009 to 2013 but remained unsatisfactory [39]. If the perception of weight status is altered and the latter is largely dependent on lifestyle, then it is likely that these characteristics are also underestimated and, for example, a parent may not care enough about their child’s diet.

It would be interesting to evaluate this aspect of other lifestyle variables (sleep and physical activity); but in this work, we do not have objective measures (e.g., the accelerometer). Further studies could implement our results.

To address the growing challenge of childhood obesity, a multifaceted approach is needed, integrating nutrition education, physical activity promotion, and family-centered interventions. Future research should further explore the role of socio-cultural factors, such as caregiver attitudes and community support systems, in enhancing the adherence to healthy dietary patterns like the MD.

## 6. Conclusions

The prevention of childhood obesity requires a synergistic effort involving an adherence to healthy dietary patterns such as the Mediterranean diet, improved caregiver awareness, and supportive community structures. By addressing misconceptions and providing tools for healthier lifestyles, it is possible to mitigate the growing prevalence of childhood obesity and its associated health risks. Adherence to the Mediterranean diet in our sample is suboptimal, highlighting the need to increase awareness among parents and caregivers, who serve as role models for their children in adopting healthier behaviors. Interestingly, the perception of their child as physically active was identified as a predictor of a better adherence to the Mediterranean diet. This finding highlights the interaction between these two lifestyle variables, which parents intuitively recognize. A better understanding of the link between parents’ perceptions and children’s habits may pave the way for more targeted and effective intervention strategies to improve health, taking into account lifestyle modification. Moreover, further longitudinal studies are needed to track the effectiveness of early interventions over time.

The successful prevention of childhood obesity requires a multi-faceted approach that incorporates families, schools, and communities. Key interventions include the following:

Caregiver Education: Programs that teach parents/caregivers to recognize their child’s weight status and adopt strategies to promote a healthier lifestyle are critical. For example, family-based interventions focusing on meal planning and limiting sugary beverages, promoting affordable alternatives.

School-Based Programs: Schools play a central role in shaping lifestyle and dietary behaviors. Implementing Mediterranean diet-based menus in school and integrating nutrition education can positively influence children’s food choices. Based on our experience, it is crucial to implement preventive measures against childhood obesity by promoting nutrition education and physical activity promotion, starting in primary schools. Early intervention helps children develop healthy eating habits, understand the importance of balanced diets, and make informed food choices. Schools play a pivotal role in shaping these behaviors, offering an ideal environment to teach the value of nutrition and encourage lifelong health-conscious practices. Schools could collaborate with healthcare professionals to provide regular, accessible feedback to parents using tools that ensure inclusivity and ease of use, providing electronic devices or apps that can inform parents but also stimulate children through educational games.

Community Support: Public health campaigns, such as those promoting local, fresh, and seasonal foods, help to normalize the MD and discourage reliance on ultra-processed foods. Awareness campaigns can amplify these efforts by promoting healthy habits through social media, public spaces, and local events. In addition, public policies must support these initiatives by investing in family-friendly spaces like parks and gyms, particularly in underserved areas.

All the approaches must be accessible, inclusive, and practical. Programs should provide free or low-cost workshops to help parents recognize their children’s weight status and adopt healthier habits in a non-stigmatizing manner, as well as educate caregivers (both parents and teachers) to recognize and address weight issues without causing guilt or stigma towards their children while fostering a supportive environment.

## Figures and Tables

**Table 1:** Parents’ demographic characteristics expressed as frequency and percentage. *n* = 95.

Answers	Frequency	Percentage
Age		
20–25	5	5.3
25–30	16	16.7
30–35	27	28.1
35–40	25	26.0
>40	22	22.9
Education level		
primary school diploma	3	3.1
middle school diploma	52	54.2
diploma	38	39.6
degree	2	2.1

**Table 2:** Devices utilization investigated by Likert scale expressed as frequency and percentage. *n* = 95.

Answer	Frequency	Percentage
1	7	7.4
2	17	25.3
3	36	37.9
4	16	16.8
5	19	20.0

**Table 3:** Parents’ perceptions about child physical activity investigated by Likert scale expressed as frequency and percentage.

How Physically Active Do You Think Your Child Is?		
Answer	Frequency	Percentage
1	1	1.1
2	3	3.2
3	18	19.1
4	19	20.2
5	54	56.4

**Table 4:** Mediterranean Diet adherence. Kid-Med scores expressed as frequency and percentage. *n* = 92, 3 respondents did not complete the questionnaire.

Kid-Med Score		
Adherence	Frequency	Percentage
Poor (Kid-Med score ≤ 3)	45	47.4
Moderate (Kid-Med score 4–7)	42	44.2
Optimal (Kid-Med score ≥8)	5	5.3

**Table 5:** Responses to Kid-Med items expressed as frequency and percentage.

Question	Answer	Frequency	Percentage
Takes fruits every day?	no	22	23.2
	yes	68	71.6
	missing	5	5.53
Has a second fruit every day?	no	67	70.5
	yes	23	24.2
	missing	5	5.3
Has fresh or cooked vegetables regularly once a day?	no	39	41.1
	yes	49	55.7
	missing	7	7.4
Has fresh or cooked vegetables more than once a day?	no	63	66.3
	yes	25	26.3
	missing	7	7.4
Consumes fish regularly (at least 2–3 times/week)	no	56	58.9
	yes	34	35.8
	missing	3	3.2
Consumes legumes at least once a week?	no	29	30.5
	yes	59	62.1
	missing	7	7.4
Consumes fast food more than once a week?	no	75	78.9
	yes	15	15.8
	missing	5	5.3
Consumes pasta or rice almost every day? (85 or more times/week)	no	29	30.5
	yes	59	62.1
	missing	7	7.4
Has cereal or grains for breakfast?	no	10	10.5
	yes	80	84.2
	missing	5	5.3
Consumes nuts regularly? (at least 2–3 times per week)	no	76	78.9
	yes	14	14.7
	missing	5	5.3
Uses olive oil at home	no	1	1.1
	yes	89	93.7
	missing	5	5.3
Skips breakfast	no	14	14.7
	yes	76	80
	missing	5	5.3
Has dairy products for breakfast?	no	28	29.5
	yes	61	64.2
	missing	5	5.3
Has commercially baked goods or pastries for breakfast?	no	61	64.2
	yes	28	29.5
	missing	5	5.3
Takes two yogurts and some cheese daily?	no	64	67.4
	yes	26	27.4
	missing	5	5.3
Takes sweets and candy several times every day?	no	41	43.2
	yes	48	50.5
	missing	5	5.3

**Table 6:** Ordinal regression. * Significance level *p* < 0.005.

	Estimation	95% CI		*p*
		inf	sup	
Constant 1	2.403	-0.283	5.089	0.080
Constant 2	5.376	2.500	8.252	0.000
Does your baby sleep more or less than 7 hours a night?	0.229	−0.629	1.088	0.601
How physically active do you think your child is?	0.575	0.098	1.053	0.018 *
Parents’ age	−0.119	−0.474	0.236	0.512
Parents’ educational level	0.076	−0.647	0.799	0.837

## Data Availability

The datasets presented in this article are not readily available because the data are part of an ongoing study.
